# Identifying Common Features in the Activation of Melanocortin-2 Receptors: Studies on the *Xenopus tropicalis* Melanocortin-2 Receptor

**DOI:** 10.3390/ijms20174166

**Published:** 2019-08-26

**Authors:** Perry E. Davis, Emily C. Wilkinson, Robert M. Dores

**Affiliations:** Department of Biological Sciences, University of Denver, Denver, CO 80210, USA

**Keywords:** *Xenopus tropicalis*, melanocortin-2 receptor, activation

## Abstract

The interaction between the pituitary hormone, adrenocorticotropin (ACTH), and melanocortin-2 receptor (MC2R) orthologs involves the H6 F7 R8 W9 and R/K15 K16 R17 R18 motifs in ACTH making contact with corresponding contact sites on MC2R. Earlier studies have localized the common HFRW binding site of all melanocortin receptors to residues in TM2, TM3, and TM6 that are located close to the extracellular space. The current study has identified residues in *Xenopus tropicalis* (xt) MC2R in TM4 (I158, F161), in EC2 (M166), and in TM5 (V172) that also are involved in activation of xtMC2R, and may be in the R/KKRR contact site of xtMC2R. These results are compared to earlier studies on the corresponding domains of human MC2R and rainbow trout MC2R in an effort to identify common features in the activation of teleost and tetrapod MC2R orthologs following stimulation with ACTH.

## 1. Introduction

For teleosts and tetrapods, the activation of the melanocortin-2 receptor (MC2R) located on glucocorticoid-synthesizing cells (i.e., interrenal cells and adrenal cortex cells) involves formation of a heterodimer between the MC2R ortholog and the accessory protein, MRAP1 [1,2,3]. The MC2R/MRAP1 heterodimer, not only facilitates the trafficking of MC2R from the endoplasmic reticulum to the plasma membrane, but also places the MC2R ortholog into the proper conformation to allow for binding of the pituitary hormone, ACTH (adrenocorticotropin; [4,5]); the only melanocortin ligand that can activate teleost and tetrapod MC2R orthologs [6].

ACTH activation of MC2R orthologs requires the coordinated interaction between two functional motifs in the hormone (i.e., H6F7R8W9 and R/K15K16R17R18), and proposed corresponding binding sites on the MC2R ortholog [7]. While all melanocortin peptides have the “melanocortin core” sequence, HFRW, that is required for activating all melanocortin receptors (i.e., MC1R, MC2R, MC3R, MC4R, MC5R), αMSH (N-acetyl-SYSMEHFRWGKPVamide) cannot activate either teleost or tetrapod MC2R orthologs [7,8] due to the absence of the tetrabasic motif (K/R15K16R17R18). Given these earlier observations by Schwyzer [7] and Mountjoy et al. [8], subsequent studies have focused on identifying first the common HFRW binding site on all melanocortin receptors, and then the unique site on MC2R orthologs required for binding the tetrabasic motif present in all vertebrate ACTH sequences [9].

**Figure 1:**
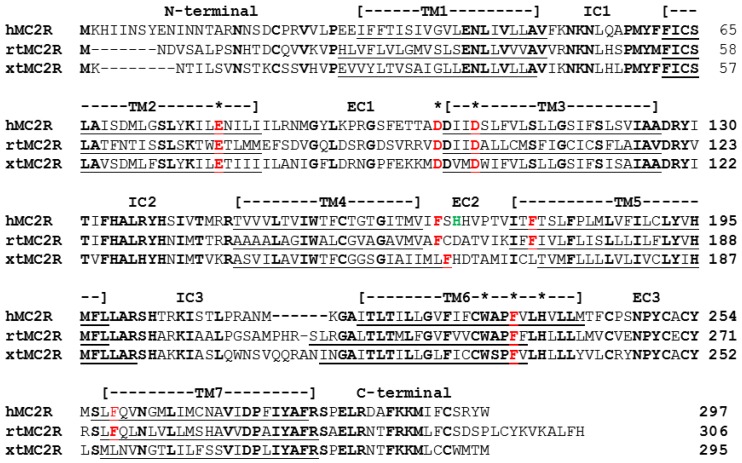
Alignment of Human, Rainbow Trout, and *Xenopus tropicalis* MC2R Orthologs. The amino acid sequences of human hMC2R (accession number: AA067714.1), rainbow trout (rtMC2R; *Onchorhynchus mykiss*; accession number: EU119870) and *Xenopus tropicalis* (xtMC2R; accession number: XP002936118.1) were aligned based on primary sequence identity. The TMHMMServer, v. 2.0-DTU (http://www.cbs.dtu.dk/services/TMHMM-2.0//) was used to identify transmembrane domains (TM; underlined). The locations of the transmembrane domains (TM), intracellular loops (IC), and extracellular loops (EC) of hMC2R are labeled. Amino acid positions in human MC2R that are proposed to be in the HFRW binding site [10] are marked with a (*). The amino acid positions in hMC2R that Chen et al. [11] identified as essential for activation are highlighted in red. H170 in EC2 that Chung et al. [12] identified as essential for activation is highlighted in green. The number of positions that are identical in all three sequences is 38%.

To localize the common HFRW binding site, Pogozheva et al. [10], used a modeling strategy and a site directed mutagenesis paradigm to identify critical amino acid positions in transmembrane (TM) domains 2, 3, and 6 of human MC4R that are required for α-MSH binding, and activation of this receptor. The latter study also observed that several of the amino acid positions in the HFRW bind site of hMC4R, were also conserved in the sequence of human MC2R (Figure 1; starred positions). Chen et al. [11] used a site directed mutagenesis approach to confirm that several of the amino acid positions in the HFRW binding site for hMC4R were also required for activation of hMC2R (Figure 1; bold red residues). These positions in hMC2R included, E80 (TM2), D103 and D107 (TM3), and F236 (TM6). In addition, Chen et al. [11] hypothesized that phenylalanine residues either on an extracellular domain (EC) or in close proximity to the extracellular space on a TM might also be important for activation of MC2R. To this end, their site directed mutagenesis analysis found that F168 (EC2), and F178 (TM5) were also important for activation. Assuming that melanocortin receptors have a barrel conformation with TM1 and TM7 in close proximity to each other, these two phenylalanine residues are clearly outside the HFRW binding site (see Figure 5A). Hence, Chen et al. [11] speculated that these residues could be in the KKRR binding site of hMC2R. Just one year later, Chung et al. [12] reported that a spontaneous mutation at H170 (EC2; Figure 1 bold green reside) resulted in a mutant form of MC2R that could traffic to the plasma membrane when expressed in a mammalian cell line, but could not be activated following stimulation with human ACTH. Collectively, these two studies pointed to the potential location of the proposed KKRR binding site in the TM4/EC2/TM5 domain of hMC2R. A more recent study of hMC2R used a single alanine substitution paradigm to evaluate the role of residues from G162 (TM4) to P183 (TM5) (Figure 1, [13]), and confirmed the importance of F168, H170, and F178 in the activation of hMC2R.

To corroborate the importance of the EC2 domain in the activation of vertebrate MC2R orthologs, a single alanine substitution study was done on the TM4/EC2/TM5 domain of a modern bony fish (Division Teleostei; *Oncyrhinchus mykiss*, *rainbow trout* (rt); [14]). This study found that alanine substitution at F160 (EC2) in rtMC2R, which aligns with F168 in the EC2 domain of hMC2R (Figure 1), disrupted activation of rtMC2R. However, since the primary sequence identity in the EC2 domain of rtMC2R and hMC2R (Figure 1) is only 11%, a consensus sequence in the EC2 required for activation was not apparent.

Since teleosts and mammals are not closely related vertebrate groups, the current study was undertaken on a MC2R ortholog of a vertebrate group, the amphibians, which occupy a phylogenetic position between the modern bony fishes and mammals [15]. The a priori assumption was that an amphibian MC2R ortholog might have features that would bridge teleost and mammalian MC2R orthologs. To this end, the current study was done on the MC2R ortholog of the amphibian, *Xenopus tropicalis* (xt).

Initial studies on xtMC2R indicated that this MC2R ortholog may be very appropriate for this type of study [16]. xtMC2R requires co-expression with the accessory protein, MRAP1, for functional expression in Chinese hamster ovary cells, and this MC2R ortholog could only be activated by ACTH, but not by any MSH-sized ligands [16,17]. However, as shown in Figure 1, the amino acid identity between the three MC2R orthologs aligned in Figure 1 is only 38% (black bold resides found at the same position in all three sequences). In addition, the amino acid sequence of the EC2 domain of xtMC2R has minimal primary sequence identity with either hMC2R or rtMC2R (Figure 1), a point that will be discussed later in this study. Assuming that a contact site for the KKRR motif of ACTH might involve residues in either TM4 or TM5 that are close to the surface, as well as one or more residues in EC2, a single alanine-substitution paradigm was used beginning with G154 (TM4) and extending to L175 (TM5) of xtMC2R to evaluate the effect of alanine substitution on the activation of xtMC2R by using a cAMP reporter gene assay. The role of residues in TM4 and TM5 that could be involved in trafficking were analyzed using a Cell Surface ELISA protocol.

## 2. Results

### 2.1. cAMP Reporter Gene Assay Analysis

While our initial studies on the activation of xtMC2R transiently transfected into CHO cells indicated that co-expression with an MRAP1 ortholog was required for activation by ACTH(1-24) [16], the MRAP1 ortholog used in that study was mouse MRAP1. Although the *X. tropicalis* genome had been sequenced, the coverage is not complete, and to date no MRAP1 sequence has been found in this database. In a later study we found that co-expressing xtMC2R with *Gallus gallus* (chicken;c) MRAP1 increased sensitivity to stimulation by hACTH(1-24) ten-fold [18] over co-expression with mouse MRAP1, and for this study xtMC2R was co-expressed with cMRAP1.

**Figure 2:**
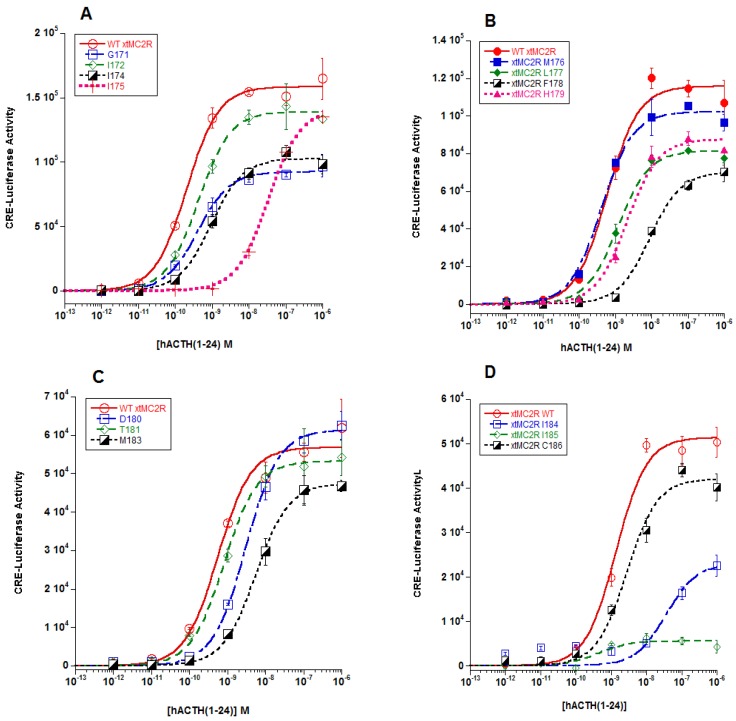

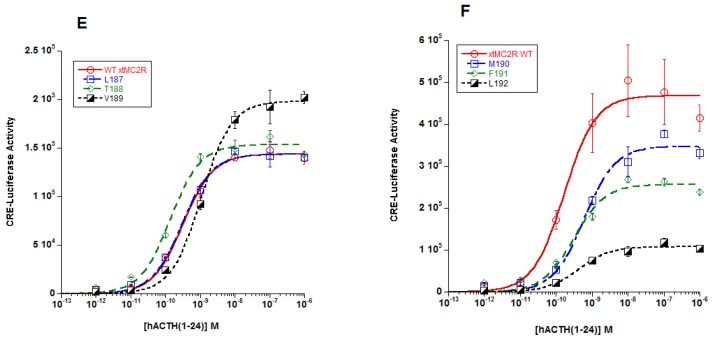
Dose response curves for xtMC2R, and single alanine mutants of xtMC2R. The wild-type receptor (xtMC2R) and single alanine mutants of xtMC2R were co-expressed with cMRAP in Chinese hamster ovary (CHO) cells as described in Materials and Methods. After two days in culture the transfected cells were stimulated with hACTH(1-24) at concentrations ranging from 10^−12^ M to 10^−6^ M. The data points are mean + SEM, *n* = 3. The data was analyzed by one-way ANOVA as described in METHODS, and EC50 values are presented in Table 1. (**A**) WT xtMC2R, G154/.A, I155/A, I157/A, I158/A. (**B**) WT xtMC2R, M159/A, L160/A, F161/A, H162/A. (**C**) WT wtMC2R, D163/A, T164/A, M165/A. (**D**) WT xtMC2R, I167/A, I168/A, C169/A. (**E**) WT wtMC2R, L170/A, T171/A, V172/A. (**F**) XT xtMC2R, M173/A, F174/A, L175/A.

The analysis of the effects of single alanine substitution in the TM4, EC2, and TM5 domains on the activation of the mutant xtMC2R constructs is shown in Figure 2A–F. In each panel the dose response curves for three to four mutant constructs co-expressed with cMRAP1 were compared to the dose response curve for the wild-type xtMC2R co-expressed with cMRAP1. The EC50 values for each assay are presented in Table 1. For the TM4 domain (i.e., G154, I155, I157, I158, M159, L160, F161), single alanine substitution at I158 resulted in nearly a 100-fold drop in activation as compared to the wild-type xtMC2R (*p* < 0.001; Figure 2A; Table 1). In addition, alanine substitution at F161 resulted in nearly a 10-fold drop in activation relative to the positive control (*p* < 0.001; Figure 2B; Table 1). For the other amino acid positions in TM4, alanine substitution did not result in a statistically significant decrease in activation as compared to wild-type MC2R (Table 1).

In the EC2 domain (i.e., H162, D163, T164, M166, I167, I168, C169, L170) single alanine substitution at residue M166 (Figure 2C) resulted in nearly a 10-fold decrease in activation relative to the positive control (*p* < 0.001; Table 1). Alanine substitution at the other amino acid positions in EC2 had no negative effect on activation as compared to the positive controls (Table 1).

Finally, for the TM5 domain (i.e., T^171^, V^172^, M^173^, F^174^, L^175^) single alanine substitution resulted in approximately a 10-fold decrease in activation for residue V^172^ (*p* < 0.001; Table 1; Figure 2E). There also was a statistically significant decrease in activation for single alanine substitution at M173 (*p* = 0.002; Table 1; Figure 2F), however the decrease was only 3-fold. Alanine substitution at the other residues in TM5 had no negative effect on activation (Table 1).

### 2.2. Cell Surface ELISA Analysis—Mutants I158/A, F161/A, V172/A, and M173/A

**Figure 3:**
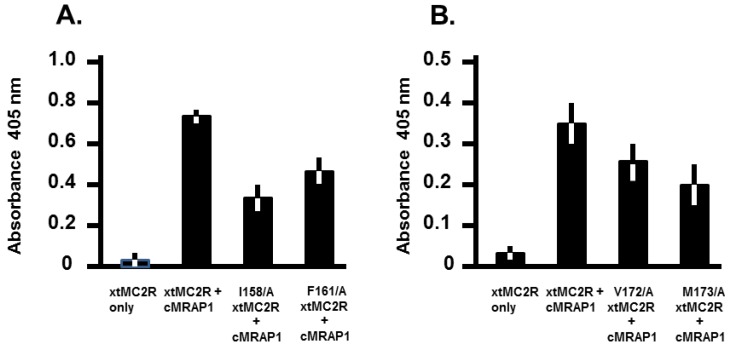
Cell Surface ELISA analysis of xtMC2R mutants I158/A, F161/A, V172/A, M173/A. xtMC2R and xtMC2R mutants, I158/A, F161/A, V172/A, M173/A were separately co-transfected with cMRAP1 in CHO cells and trafficking of the receptor to the plasma membrane was evaluated by cell surface ELISA as described in Material and Methods. (**A**) Negative control, xtMC2R expressed alone; positive control, xtMC2R + cMRAP1, mutants analyzed: I158/A xtMC2R + cMRAP1, and F161/A xtMC2R + cMRAP1. Note that there was a significant increase in the trafficking when xtMC2R was co-expressed with cMRAP1 as compared to xtMC2R expressed alone (*p* < 0.001). (**B**) Negative control, xtMC2R expressed alone; positive control, xtMC2R + cMRAP1, mutants analyzed: V172/A xtMC2R + cMRAP1, and M^173^/A xtMC2R + cMRAP1. Note that there was a significant increase in the trafficking when xtMC2R was co-expressed with cMRAP1 as compared to xtMC2R expressed alone (*p* < 0.001). Data points are mean + SEM; *n* = 3.

Since the trafficking of MC2R orthologs to the plasma membrane involves interaction between a TM domain on the receptor, and the TM domain of MRAP1 [2], it is possible that a drop in activation observed for alanine substitution at residues I158 and F161 in TM4, and V172 and M173 in TM5 may have been the result of interference with the trafficking of the mutant receptor to the plasma membrane. To evaluate this possibility, a cell surface ELISA analysis was done. As shown in Figure 3A, the trafficking of the I158/A mutant to the plasma membrane was significantly decreased (*p* > 0.001) relative to the positive control. However, the trafficking of the F161/A mutant was not statistically different from the trafficking of the positive control (*p* = 0.06). The same analysis was done for mutants V172/A and M173/A from TM5, and the trafficking of either mutant was not statistically different from the positive control (Figure 3B; *p* = 0.93 and 0.99, respectively).

### 2.3. Western Blot Analysis: Mutants I158/A, F161/A, M166/A, V172/A

**Figure 4:**
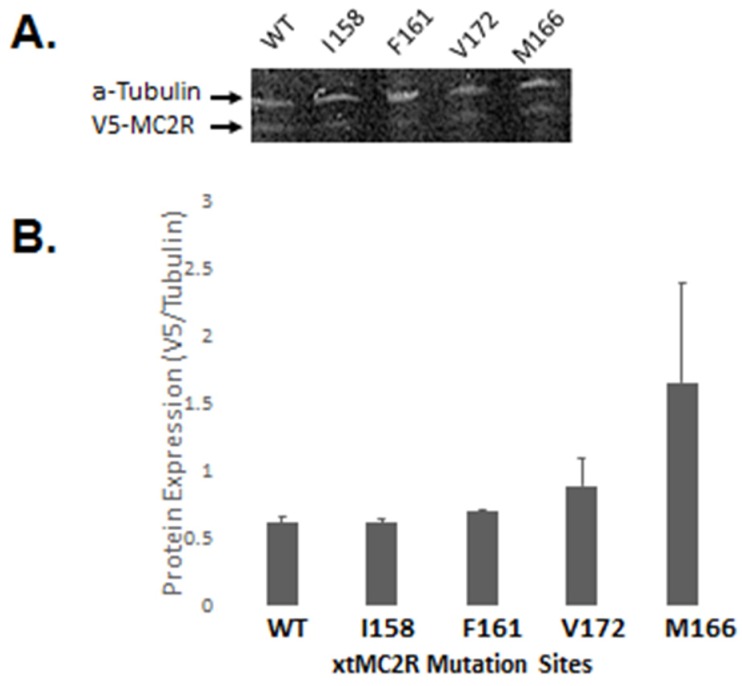
Western blot analysis - protein expression of xt MC2R single alanine mutants, I158/A, F161/A, V172/A, and M166/A. (**A**) This figure shows the overall protein expression of WT and single alanine mutants xMC2R I158/A, F161/A, V172/A, and M166/A transiently transfected in CHO cells with cMRAP. Whole cell lysates were prepared in in RIPA buffer and 500 µg total protein was loaded into a 4–20% Mini-PROTEAN^®^ TGX™ Precast Protein Gels (BioRad, Herculus, CA, USA) to perform western blot analysis. a-Tubulin was used as a loading control. (**B**) This figure shows the quantification of overall WT hMC2R or mutant expression. V5 tagged receptor expression was normalized to a-Tubulin and compared using a one-way ANOVA.

To evaluate whether alanine substitution might have a negative effect on the transcription of the mutant constructs, four of the xtMC2R/A mutant constructs were selected for the western blot experiment [I158/A and F161/A (TM4), V172/A (TM5), M166/A (EC2)]. Each mutant construct and the wild-type xtMC2R cDNA had an N-terminal V5 epitope tag. For this analysis, each construct was separately transfected at the same concentration in CHO cells, and after five days in culture the transfected cells were harvested, and the cell extracts were analyzed by western blot (Figure 4A). While all five cDNA generated a signal (Figure 4A), densitometry reading (Figure 4B) indicated that the expression levels for the four mutant constructs were not statistically different from the expression level for the wild-type xtMC2R. Hence, the decrease in the trafficking of the I158/A mutant, and the decrease in activation for the F161/A mutant, the M166/A mutant, and the V172/A mutant do not appear to an expression artifact.

## 3. Discussion

MC2R is perhaps the most complex and enigmatic member of the melanocortin receptor gene family. This receptor plays a pivotal role in the hypothalamus/pituitary/adrenal/interrenal (HPA/HPI) Axis by inducing glucocorticoid synthesis from either adrenal cortex cells (mammals and reptiles) or interrenal cells (amphibians, bony fishes, cartilaginous fishes; [19,20]). As a result, this neuroendocrine circuit helps to re-establishing homeostasis after a chronic stress event in both teleosts and tetrapods [19]. Given the importance of the HPA/HPI axis, it would be reasonable to predict that selection pressures would favor conservation of the primary sequences of ACTH, the ligand that binds to the glucocorticoid producing cells, and the ACTH receptor (i.e., MC2R). As predicted, the primary sequence of ACTH, within the minimal sequence required for full activation at target cells (i.e., ACTH(1-24); [7]), is 89% for ACTH(1-24) sequences from a broad taxonomic spectrum (i.e., cartilaginous fishes to mammals; [9]). All vertebrate ACTH sequences have the canonical motif, H6F7R8W9, that is required for activating all melanocortin receptors [20], and the R/K15K16R17R18 motif that is required for facilitating the activation of all teleost and tetrapod MC2R orthologs [7,20,21].

Given the observations for ACTH, the status of teleost and tetrapod MC2R orthologs is surprising. The primary sequence identity for the three bony vertebrate MC2R orthologs presented in Figure 1 is only 38%. While this level of primary sequence conservation may seem reasonable for species of vertebrates that last shared a common ancestor over 400 million years ago [22], a recent analysis of the primary sequence identity between a cartilaginous fish (stingray) MC5R ortholog and a mammalian (human) MC5R ortholog was found to be 55% [23]. In mammals, MC5R plays a role in sebaceous gland secretion [24]; whereas in non-mammalian vertebrates including stingrays, the function(s) and the selection pressures on MC5R orthologs are unknown. Clearly, melanocortin receptors paralogs have not been evolving at the same rate during the radiation of the gnathostomes. Given the physiological importance of the HPA/HPI axis, it seems remarkable that in spite of the accumulation of point mutations, teleost and tetrapod MC2R orthologs have retained functionality, and share a number of unique physiological features not seen in the other melanocortin receptors.

For example, the MC2R orthologs of teleosts and tetrapods, unlike the other paralogs in this gene family (i.e., MC1R, MC3R, MC4R, and MC5R), can only be activated by ACTH, and not by any of the MSH-sized melanocortin peptides derives from POMC [7,8,21]. In fact, it appears that MSH-sized melanocortin peptides cannot even bind to the “ACTH” receptor (i.e., MC2R; [25]). The inability to bind αMSH is an apparent enigma given that MC2R orthologs share an HFRW binding pocket in common with all the other teleost and tetrapod melanocortin receptors [10,26,27]. In addition, teleost and tetrapod MC2R orthologs have intracellular trafficking restrictions which mandate that the receptor form a heterodimer with the accessory protein MRAP1 to facilitate unimpeded movement of the receptor from the endoplasmic reticulum to the plasma membrane [4,5]. Finally, for activation at the plasma membrane, MC2R must be in a complex with MRAP1for the ACTH binding event to occur [1,2]. Thus, the singular feature that sets teleost and tetrapod MC2R orthologs apart from the other members of the melanocortin receptor gene family is the obligatory interaction with MRAP1; an interaction that may have begun prior to the radiation of the ancestral gnathostomes [13].

**Figure 5:**
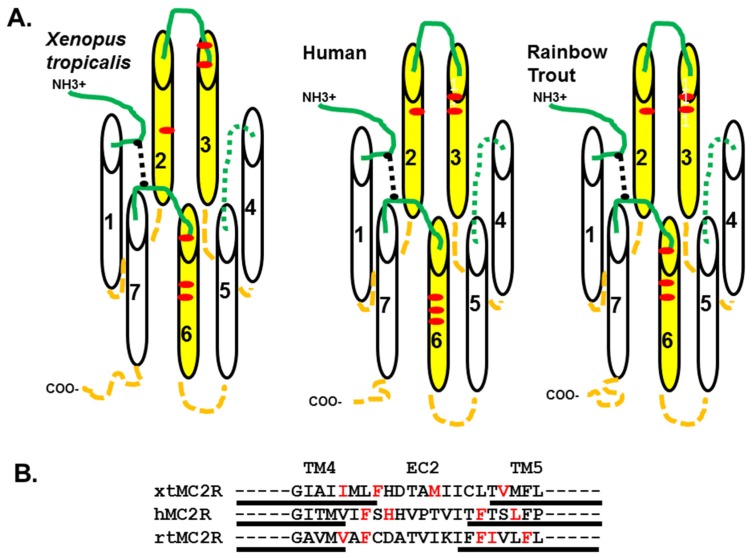
Schematic representation of human, rainbow trout, and *X. tropicalis* MC2R. The diagrams for the proposed positioning of transmembrane domains in the MC2R orthologs for *X. tropicalis*, human, and rainbow trout is based on the alignments presented in Figure 1, and the presence of a disulfide bridge between cysteine residues in TM1 and TM7 that is essential for functional activation of the receptor. The transmembrane domains (TM) are represented as numbered cylinders. TMs shaded in yellow form the proposed HFRW binding site. Extracellular domains are green lines. The N-terminal domain is capped with “NH3+.” Extracellular Loop 2 is shown as a green dotted line. Intracellular domains are orange dashed lines. The C-terminal domain is capped by “COO-.” The disulfide bridge between the TM1 and TM7 [32] is a black dotted line. In each diagram, critical residues associated with the proposed HFRW binding site are a red dot. In *X. tropicalis* MC2R the residues that may play a role in the HFRW binding pocket are E72 (TM2), D95 (EC1) and D99 (TM3), and W230, F233, and H236 (TM6). In human MC2R the residues that play a role in the HFRW binding pocket are E80 (TM2), D103 (EC1), D107 (TM3), W233, F236, and H239 (TM6). For rainbow trout MC2R the residues that may play a role in the HFRW binding pocket are E73 (TM2), D96 (EC1), D100 (TM3), W231, F234, and H237 (TM6).

Since α-MSH lacks the R/KKRR motif, and as a result cannot activate teleost or tetrapod MC2R orthologs, it would appear that the activation mechanism for MC2R orthologs may involve a two-step process in which the R/KKRR motif of ACTH docks with the receptor, and this event causes a conformation change which exposes a site on the receptor for the binding of the HFRW motif of ACTH. It would be the later proposed binding event that leads to the activation of the G-protein associated with the receptor [28]. Based on the predications made with the TMHMMServer, v. 2.0-DTU program on the location of transmembrane domains in the three MC2R orthologs in Figure 1, a schematic representation of the three receptors is presented in Figure 5A. Note that six of the critical residues that formed the HFRW binding site in hMC4R [10] are in nearly the same position in or near the TM2 and TM3 domain, and in or near the TM6 domain of the three MC2R orthologs. Malik et al. [29] observed that alanine substitution at E80 rendered the mutant hMC2R inactive to ACTH stimulation, but had no effect on the trafficking of the mutant MC2R when co-expressed with MRAP1. The same outcome would be expected for E172 in xtMC2R and E172 in rtMC2R. While site-directed mutagenesis experiments are needed to verify the role of E172 in the activation of xtMC2R, the models presented in Figure 5A point to EC2 as another potential contact site for ACTH interaction with MC2R as previously proposed by Chen et al. [11].

The focus, then, of this study was to evaluate the role that residues in the TM4/EC2/TM5 domain of xtMC2R may play in the activation of this receptor. The results from previous studies using a single-alanine substitution paradigm for hMC2R [13] and rtMC2R [14] are shown in Figure 5B. The current study was done on the corresponding region of xtMC2R with the following results. Single alanine substitution at I158, F161, two residues in TM4 located close to extracellular space, M166 in EC2, and V172 in TM5 also located near the extracellular space all resulted in a drop in sensitivity to stimulation by hACTH(1-24) of 10 fold or more in the cAMP reporter gene assay analysis (Figure 2; Table 1; Figure 5B). The location of these residues would be consistent with a shallow hydrophilic pocket at the TM4/EC2/TM5 domain of xtMC2R. These results are comparable to our earlier studies on the TM4/EC2/TM5 domain of hMC2R [13] and the TM4/EC2/TM5 domain of rtMC2R ([14]; Figure 5B). For all three species, a limited number of amino acid positions (4 to 5) were negatively impacted by single alanine substitution. What is perplexing about these results (Figure 5B) is that a consensus sequence for modulating activation of the three MC2R orthologs within this domain is not apparent. The assumption is that the EC2 domain and perhaps a portion of TM5 form the putative R/RKRR binding site for ACTH. Hence, it might be expected that this site would be rich in acidic amino acids that presumably would ionically interact with the R/KKRR motif of ACTH, but this was not the case. However, there are a few aromatic amino acids in this region such as phenylalanine, and alanine substitution at these residues consistently lowered sensitivity to stimulation by ACTH(1-24). 

In the alanine substitution study for rtMC2R [14], a cell surface ELISA analysis indicated that V158, F171, and F175 (highlighted in Figure 5B) did not have any negative effect on trafficking. The same analysis was done for I158, F161 in TM4 of xtMC2R, and V172 and M173in TM5 of xtMC2R (Figure 3). The residues in TM5 had no negative effect on trafficking (Figure 3B), and neither did residue F161 in TM4 (Figure 3A). However, alanine substitution at residue I158 in TM4 apparently reduced trafficking of the receptor to the plasma membrane by nearly 50%. While the later observation is preliminary, it appears that TM4 could be the contact site for the TM of MRAP1; an interaction that would facilitate trafficking. Now an in-depth analysis of the other residues in TM4 will need to be done to clarify the role of TM4 in trafficking. Since the three studies summarized in Figure 5B, all required co-expression with an MRAP1 ortholog, perhaps the critical question with respect to the role of TM4, EC2, and TM5 in the activation of MC2R should refocus on establishing the actual contact site between MRAP1 and MC2R when the MC2R/MRAP1 heterodimer forms at the ER [3,4].

As noted previously, the interaction between MRAP1 and MC2R involves both trafficking of the receptor, and activation of the receptor [3,4]. The trafficking function for MRAP1 requires that the TM of MRAP1 make contact with some TM domain in MC2R [2]. The activation function associated with MRAP1 resides in an amino acid motif (activation motif) in the N-terminal of the MRAP1 homodimer [3,4]. Neither corresponding domains in hMC2R (trafficking or activation) had been identified prior to 2010. Initial attempts at resolving these issues have used a chimeric receptor paradigm [30,31], and collectively these studies point to a role for TM4 in trafficking of hMC2R. In addition, the results from both studies could be explained by assuming that formation of the heterodimer between MRAP1 and MC2R results in a conformation change in the receptor which places the R/KKRR binding site (presumably EC2) in the proper conformation for the activation process to begin.

In a more recent study, Malik et al. [29] found definitive evidence that at the plasma membrane, the activation motif [3,4] on the N-terminal domain of MRAP1 that is facing the extracellular space makes contact with an extracellular domain on hMC2R to facilitate activation. Since MRAP1 is a homodimer with reverse topology [2], when at the plasma membrane the homodimer would have a N-terminal domain of one monomer in the homodimer facing the extracellular space and one monomer with the N-terminal domain facing the cytosol. Since both N-terminal domains carry the activation motif, interaction with MC2R potentially could be through either an extracellular loop, or an intracellular loop. The later study eliminated the intracellular loops as the contact site, but did not identify the extracellular domain making contact with the activation motif of MRAP1. The implications of the Malik et al. [29] study are very relevant to the current study. While the current study, and the previous alanine-substitution studies [11,13,14] have focused on the role of EC2 in the activation process, it may be more expedient to first identify the extracellular domain in MC2R that makes contact with the activation motif on the N-terminal of MRAP1. That contact site should then point to the adjacent transmembrane domain(s) in MC2R making contact with the transmembrane domain of MRAP1 to facilitate trafficking. With that information in hand the role of EC2 in the activation process may be clarified.

## 4. Material and Methods

### 4.1. DNA Constructs

All cDNA constructs of xtMC2R were synthesized by GenScript (Piscataway, NJ, USA). Each construct was designed with an N-terminal V5 epitope tag, and each construct was inserted into a pcDNA3.1+ vector. The xtMC2R cDNA constructs included wild-type xtMC2R (accession number: XP002936118.1) and single alanine mutants of xtMC2R (Figure 1: G154 to L175 excluding positions 156, and 165 where alanine residues were located. GenScript also synthesized the Gallus gallus (chicken; c) cMRAP1 (accession number: XR_001470382.1) cDNA construct, and this construct was also inserted into a pcDNA3.1+ vector. The cAMP reporter gene construct CRE-Luciferase [33] was provided by Dr. Patricia Hinkle (University of Rochester, NY, USA).

### 4.2. Tissue Culture

For this project, the cDNAs were expressed in Chinese hamster ovary (CHO) cells (ATCC, Manassas, VA, USA) as described in Liang et al. [16]. The CHO cells were grown to confluence in Kaighn’s Modification of Ham’s F12K media (ATCC). The incubation media was supplemented with 10% fetal bovine serum, 100 unit/mL penicillin, 100 μg/mL streptomycin, 100 μg/mL normocin, and maintained in a humidified incubator with 95% air and 5% CO_2_ at 37 °C.

### 4.3. ACTH Analog Peptide

Human (h) ACTH(1-24) was purchased from New England Peptides (Gardiner, MA, USA). For the cAMP-reporter gene assay, hACTH(1-24) was used to stimulate transfected cells at concentrations ranging from 10–12 M to 10–6 M.

### 4.4. cAMP-Reporter Gene Assay

For the cAMP-reporter gene assay, 3.0 × 10^6^ CHO cells were transiently transfected with either the xtMCR cDNA construct or one of the single alanine xtMC2R mutants, a cMRAP1 cDNA, and the CRE-Luciferase cDNA construct following the protocol described in Liang et al., [16]. The transfections were performed using the Amaxa Cell Line Nucleofector II system (Lonza, Portsmouth, NH, USA), and utilized the Solution T transfection kit. Transfected cells were seeded in a white 96-well plate at a final density of 1 × 10^5^ cells/well. Stimulation with concentrations of hACTH(1-24) in serum-free CHO media was done 2 days after transfection and plating, and the hormone stimulation period was 4 hours at 37 °C. The hormone stimulation was terminated by adding Bright GLO (Promega, Madison, WI, USA), the luciferase substrate reagent. After a 5-minute incubation period at room temperature, luminescence was immediately measured using a Bio-Tek Synergy HTX plate reader (Winooski, VT, USA). To determine the background levels of cAMP production, a set of transfected CHO cells were stimulated with serum free CHO media only (no ACTH) for the four-hour incubation period, and the average background luminescence reading for these control wells was subtracted from the ligand-stimulated luminescence readings. The dose response curves for the stimulated cells were analyzed using the Michaelis–Menton equation to obtain EC50 values. All assays were done in triplicate. The data were plotted using Kaleidograph software (www.synergy.com).

### 4.5. Cell Surface ELISA

CHO cells were transfected with 0.1 μg/mL wildtype or single alanine mutant xtMC2R and 0.1 μg/mL cMRAP1 using jetPRIME transfection reagent (Polyplus transfection SA, Illkirch, France). Cells were grown for 48 h at 37 °C and then incubated on ice. Cells were surface-labeled with anti-V5 antibody (1:1000, Rockland Immunochemicals, Limerick, PA, USA) and then fixed in 4% paraformaldehyde, pH 7.2 (Sigma, St. Louis, MO, USA). Fixed cells were washed in 1x phosphate-buffered saline and incubated at room temperature with HRP-conjugated goat-anti-rabbit secondary antibody (Sigma, St. Louis, MO, USA). Cells were then washed and treated with 1-step ABTS (Thermo-Fisher Scientific, Watham, MA, USA). Absorbance was measured at 405 nm using a Bio-Tek Synergy HTX plate reader (Winooski, VT, USA). The analyses were done in triplicate. For each analysis the negative control was xtMC2R transfected alone, and the positive control xtMC2R co-transfected with cMRAP1.

### 4.6. Western Blot Protocol

CHO cells were separately transfected with either wild-type xtMC2R or xtMC2R mutant I158/A, F161/A (TM4), V172/A (TM5) or M166/A (EC2) and each can was co-expressed with cMRAP for 5 days in advance of cell lysis. Cells were homogenized in RIPA buffer and sonicated to collect total protein. Sample protein levels were then measured by nano-spectrophotometry and analyzed by poly-acrylamide gel electrophoresis. A 4% to 20% Mini-PROTEAN® TGX™ Precast Protein Acrylamide Gels (Biorad, Hercules, CA, USA), and then samples were transferred to a nitrocellulose membrane (Genesee Scientific; San Diego, CA, USA). Membrane was blotted with anti-V5 antibody (Rockland Immunochemicals, Limerick, PA, USA) at a final concentration of 1:500, and anti-alpha-tubulin from Developmental Studies Hybridoma Bank (University of Iowa) at a final concentration of 1:500. Membrane was scanned using a Protein Simple FluorChem system (San Jose, CA, USA) and analyzed using Fiji; an open-source platform for biological-image analysis. The Western analysis was done in triplicate.

### 4.7. Statistical Analysis

Data points are expressed as the mean + standard error of the mean (*n* = 3). For the cAMP reporter gene assay, statistical differences between the EC50 value of the xtMCR positive control and the xtMCR single alanine mutants were evaluated using one-way ANOVA followed by Tukey’s multi-comparison test using GraphPad Prism 2 (GraphPad Software Inc, La Jolla, CA, USA) for equal variance. Significance was set at *p* ≤ 0.05. For the Cell Surface ELISA assay, statistical differences were also evaluated using on-way ANOVA followed by Tukey’s multi-comparison test (GraphPAD). The western blot analysis was done in triplicate, and the intensity of the blots was analysis using a one-way ANOVA.

## Figures and Tables

**Table 1:** EC50 Values for xtMC2R Single Alanine Mutants.

EC50			*P*-Value
Wt	xtMC2R	2.0 × 10^−10^ M ± 2.4 × 10^−11^	−
G154	(TM4)	4.1 × 10^−^^10^ M ± 4.8 × 10^−11^	0.99
I155	(TM4)	4.2 × 10^−10^ M ± 4.4 × 10^−11^	0.99
I157	(TM4)	9.4 × 10^−10^ M ± 1.3 × 10^−10^	0.99
I158	(TM4)	3.4 × 10^−8^ M ± 2.3 × 10^−9^	˂0.001
Wt	xtMC2R	1.2 × 10^−^^10^ M ± 1.4 × 10^−^^10^	−
M159	(TM4)	1.0 × 10^−^^10^ M ± 6.4 × 10^−^^11^	0.99
L160	(TM4)	1.2 × 10^−^^9^ M ± 1.6 × 10^−^^10^	0.99
F161	(TM4)	8.8 × 10^−^^9^ M ± 1.1 × 10^−^^9^	˂0.001
H162	(EC2)	2.1 × 10^−^^9^ M ± 4.1 × 10^−^^10^	0.76
Wt	xtMC2R	5.2 × 10^−^^10^ M ± 1.3 × 10^−^^10^	−
D163	(EC2)	1.2 × 10^−^^10^ M ± 1.4 × 10^−^^10^	0.17
T164	(EC2)	8.1 × 10^−^^10^ M ± 7.8 × 10^−^^11^	0.99
M166	(EC2)	5.5 × 10^−^^9^ M ± 4.0 × 10^−^^10^	˂0.001
Wt	xtMC2R	1.5 × 10^−^^9^ M ± 2.6 × 10^−^^10^	−
I167	(EC2)	3.6 × 10^−^^8^ M ± 2.5 × 10^−^^8^	0.99
I168	(EC2)	4.2 × 10^−^^10^ M ± 3.4 × 10^−^^10^	0.21
C169	(EC2)	2.8 × 10^−^^9^ M ± 6.5 × 10^−^^10^	0.99
Wt	xtMC2R	3.2 × 10^−^^10^ M ± 3.6 × 10^−^^11^	−
L170	(EC2)	3.0 × 10^−^^10^ M ± 4.3 × 10^−^^11^	0.99
T171	(TM5)	1.4 × 10^−^^10^ M ± 2.8 × 10^−10^	0.21
V172	(TM5)	1.1 × 10^−^^9^ M ± 1.0 × 10^−10^	˂0.001
Wt	xtMC2R	1.6 × 10^−^^10^ M ± 4.5 × 10^−^^11^	−
M173	(TM5)	5.9 × 10^−^^10^ M ± 1.4 × 10^−^^10^	0.002
F174	(TM5)	3.1 × 10^−^^10^ M ± 1.0 × 10^−10^	0.06
L175	(TM5)	4.3 × 10^−^^10^ M ± 1.1 × 10^−^^10^	0.05
